# The Dynamics of Balanced Spiking Neuronal Networks Under Poisson Drive Is Not Chaotic

**DOI:** 10.3389/fncom.2018.00047

**Published:** 2018-06-28

**Authors:** Qing-long L. Gu, Zhong-qi K. Tian, Gregor Kovačič, Douglas Zhou, David Cai

**Affiliations:** ^1^School of Mathematical Sciences, MOE-LSC, Institute of Natural Sciences, Shanghai Jiao Tong University, Shanghai, China; ^2^Mathematical Sciences Department, Rensselaer Polytechnic Institute, Troy, NY, United States; ^3^Courant Institute of Mathematical Sciences, Center for Neural Science, New York University, New York, NY, United States; ^4^NYUAD Institute, New York University Abu Dhabi, Abu Dhabi, United Arab Emirates

**Keywords:** balanced state, irregular activity, chaotic dynamics, delta-pulse coupling, largest Lyapunov exponent

## Abstract

Some previous studies have shown that chaotic dynamics in the balanced state, i.e., one with balanced excitatory and inhibitory inputs into cortical neurons, is the underlying mechanism for the irregularity of neural activity. In this work, we focus on networks of current-based integrate-and-fire neurons with delta-pulse coupling. While we show that the balanced state robustly persists in this system within a broad range of parameters, we mathematically prove that the largest Lyapunov exponent of this type of neuronal networks is negative. Therefore, the irregular firing activity can exist in the system without the chaotic dynamics. That is the irregularity of balanced neuronal networks need not arise from chaos.

## 1. Introduction

Neural spiking activity in the brain is highly irregular (Britten et al., 1993; Shadlen and Newsome, 1998; Compte et al., 2003; London et al., 2010). It is believed that the irregularity of the spiking activity can reflect an underlying rich coding structure for information processing (Hertz and Prügel-Bennett, 1996; Gütig and Sompolinsky, 2006; Sussillo and Abbott, 2009; Monteforte and Wolf, 2012). This viewpoint naturally leads to the investigation of the origin of the irregular neuronal activity. A number of theoretical studies have postulated that a balance between excitatory and inhibitory inputs into an individual neuron can give rise to irregular activity (van Vreeswijk and Sompolinsky, 1996; Troyer and Miller, 1997; Vreeswijk and Sompolinsky, 1998; Vogels and Abbott, 2005; Miura et al., 2007). The idea behind the theory of balanced networks is that the excitatory and inhibitory components of inputs nearly cancel each other, and the neuronal firing activity is driven by strong fluctuations that intermittently interrupt this cancellation. Consistent with the hypothesized scenario, balanced synaptic inputs have been observed in slices of the ferret prefrontal and occipital cortex (Shu et al., 2003). Moreover, it has also been found that, *in vivo* studies, the balanced excitation and inhibition in ferrets' prefrontal cortex can substantially influence the neuronal activity (Haider et al., 2006).

It has been shown theoretically that small perturbations of the balanced state in a network with *binary* neurons grow exponentially, indicating the chaotic nature of the balanced activity (Vreeswijk and Sompolinsky, 1998). Some studies suggest that highly irregular activity in the balanced state originates from chaotic network dynamics (Vogels et al., 2005; Wallace et al., 2013; Ostojic, 2014). Albeit not specifically addressing the source of irregular activity in a balanced state, studies exist that have drawn the opposite conclusions in some special situations. For example, numerical simulations of neural networks consisting of pulse-coupled spiking neurons of only inhibitory type can display irregular activity in a dynamical state with a negative Lyapunov exponent (Zillmer et al., 2006; Jahnke et al., 2008; Monteforte and Wolf, 2012). Meanwhile, in the limit of fast synaptic response, any generic trajectory was shown to be asymptotically stable in inhibition-dominated networks (only a small fraction of connections can be excitatory) with the inhomogeneous delay distribution and strong coupling (Jahnke et al., 2009). Therefore, the question of whether the irregularity in a balanced state arises from chaos in a neuronal network with both excitatory and inhibitory neurons under more realistic Poisson drive, remains an important issue to be further clarified.

In this work, we first numerically show that, over a broad range of parameters, the balanced state can exist in current-based integrate-and-fire (I&F) neuronal networks consisting of both excitatory and inhibitory neurons with delta-pulse coupling currents and pulse-like external inputs. We then mathematically prove that, driven by any point process in time—not limited to Poisson point processes, the current-based I&F neuronal networks with delta-pulse interactions cannot exhibit chaotic dynamics. In fact, two nearby trajectories of such a network generically coalesce after a finite time regardless of whether the dynamics is in a balanced state or not. Our results demonstrate that in the delta-pulse coupled, current-based I&F system the irregular activity of the balanced state is not a consequence of a chaotic dynamical state. Our proof remains valid when the system possesses only excitatory or inhibitory population. This conclusion extends the previous results (Jin, 2002; Zillmer et al., 2006; Jahnke et al., 2009) that dynamics of the strongly inhibition-dominated networks are stable. By our analysis, stable dynamics with the irregular firing activity can occur in the delta-pulse coupled, current-based I&F neuronal network of any size.

## 2. Materials and methods

### 2.1. I&F model

We model neurons as integrate-and-fire (I&F) units (Dayan and Abbott, 2001; Newhall et al., 2010; Zhou et al., 2010). The governing equation for the membrane potential $v_{i}^{k}$ of the *i*th neuron in the *k*th population is

(1) $$\begin{array}{r} \frac{dv_{i}^{k}}{dt}=-g_{L}\left(v_{i}^{k}-\epsilon_{R}^{k}\right)+I_{i}^{k}\left(t\right), \end{array}$$

where *g*_*L*_ denotes the leakage conductance, $\epsilon_{R}^{k}$ is the resting voltage of the *k*th population and $I_{i}^{k}\left(t\right)$ is the corresponding input current (*k* = *E, I*). The voltage $v_{i}^{k}$ evolves according to Equation (1) when $v_{i}^{k}\leq \epsilon_{T}^{k}$, where $\epsilon_{T}^{k}$ is the threshold of the *k*th population. When $v_{i}^{k}$ crosses $\epsilon_{T}^{k}$, the neuron spikes, and then $v_{i}^{k}$ is reset to $\epsilon_{R}^{k}$. Upon resetting, $v_{i}^{k}$ is immediately governed by Equation (1) again. In simulation, $g_{L}=50\;{\text{s}}^{-1}$ corresponds to the membrane time constant 20 ms. The dimensionless values used in simulations are $\epsilon_{R}^{E}=\epsilon_{R}^{I}=0.0$, $\epsilon_{T}^{E}=1.0$ and $\epsilon_{T}^{I}=0.7$, which correspond to the parameters in Vreeswijk and Sompolinsky (1998).

The instantaneous current projecting into the *i*th neuron in the *k*th population,

(2) $$\begin{array}{r} I_{i}^{k}\left(t\right)=I_{i}^{kE}\left(t\right)+I_{i}^{kI}\left(t\right), \end{array}$$

consists of two terms, where $I_{i}^{kE}\left(t\right)=f^{k}\underset{s}{\sum }\delta \left(t-\zeta_{is}^{k}\right)+J^{kE}\overset{N^{E}}{\underset{j=1}{\sum }}C_{ij}^{kE}\underset{s}{\sum }\delta \left(t-\tau_{js}^{E}\right)$ is the excitatory input, $I_{i}^{kI}\left(t\right)=-J^{kI}\overset{N^{I}}{\underset{j=1}{\sum }}C_{ij}^{kI}\underset{s}{\sum }\delta \left(t-\tau_{js}^{I}\right)$ is the inhibitory input, δ(·) is the Dirac delta function, *f*^*k*^ is the strength of the external input, and *J*^*kl*^ is the coupling strength from the *l*th population to the *k*th population, *k, l* = *E, I*. The coupling constant $C_{ij}^{kl}=0\text{ or }1$ is an element of the adjacency matrix of the network. It describes the connection from the *j*th neuron in the *l*th population to the *i*th neuron in the *k*th population. The first term in $I_{i}^{kE}\left(t\right)$ corresponds to the current arriving from the external input. The spike-time sequence, $\left\{\zeta_{is}^{k},s=1,2,\ldots \right\}$, corresponds to the external input into the *i*th neuron in the *k*th population. At the arrival time $\zeta_{is}^{k}$ of the *s*th spike, the voltage of the *i*th neuron in the *k*th population jumps by the amount of *f*^*k*^. In the simulations below, we use Poisson trains for the external inputs. The second term in $I_{i}^{kE}\left(t\right)$ and the term in $I_{i}^{kI}\left(t\right)$ correspond to the presynaptic input from neurons of the excitatory and inhibitory populations in the network, respectively, where $\tau_{js}^{k}$ is the arrival time of the *s*th spike from the *j*th neuron in the *k*th population for *k* = *E, I*.

In our simulation, each neuron in the network has, on average, *K* excitatory and *K* inhibitory presynaptic neurons. Because each neuron may receive a large number of synapses in the cortex (Peters, 1987; Braitenberg and Schuz, 1998), and the connection between cortical neurons is often sparse with low connection probability (Holmgren et al., 2003), we choose *K* to be sufficiently large but much smaller than the total number of neurons in the network. Experimentally, for example, it is observed that cells in the primary visual cortex of adult cats fire much more irregularly than cells *in vitro* when they are both stimulated by injecting direct current through the electrode. Therefore, there is a substantial influence of fluctuations of synaptic inputs on the irregular activity (Holt et al., 1996). To capture the effect of fluctuations as observed in the experiment, we follow the balanced network theory to set the scaling of the coupling strength *J*^*kl*^ to be of order $1/\sqrt{K}$, thus leading to the scaling of fluctuations in the total synaptic inputs as of order 1. As a consequence, the fluctuations persist in the large-*K* limit (van Vreeswijk and Sompolinsky, 1996; Vreeswijk and Sompolinsky, 1998; Vogels and Abbott, 2005).

The connection from the *j*th neuron in the *l*th population to the *i*th neuron in the *k*th population $C_{ij}^{kl}$ in our simulation follows a Bernoulli distribution, i.e., the probability $P\left(C_{ij}^{kl}=1\right)=K/N^{l}$ and $P\left(C_{ij}^{kl}=0\right)=1-K/N^{l}$, where *N*^*l*^ is the total number of the *l*th population, *k, l* = *E, I*. The parameter values in our simulations are as follows: *N*^*E*^ = 32000, *N*^*I*^ = 8000, *K* = 400, $J^{EE}=J^{IE}=1.0/\sqrt{K}$, $J^{II}=1.8/\sqrt{K}$, $J^{EI}=2.0/\sqrt{K}$, $f^{E}=1.0/\sqrt{K}$, $f^{I}=0.8/\sqrt{K}$, and the external inputs are Poisson processes with rate ν^*E*^ = ν^*I*^ = ν^0^*K*, where ν^0^ controls the magnitude of Poisson rate.

Simulations of the neuronal network model are carried out to the machine accuracy using the event-driven algorithm (Brette et al., 2007). The event-driven algorithm for a general point process as external inputs proceeds by generating the time of the next external spike. Some neurons in the network receive this spike. Upon receiving an external spike, these neurons' voltages increase by the amount of *f*^*E*^ for the excitatory population and *f*^*I*^ for the inhibitory population. If some of them have their voltages exceed the threshold, they fire. Their voltages are held at the reset voltage, and the voltages of their postsynaptic neurons are instantaneously increased (for excitatory inputs) or decreased (for inhibitory inputs). It is possible that the voltages of these postsynaptic neurons may now be above threshold as well, then, these neurons also fire. Their voltages are held at the reset voltage, while their postsynaptic neurons' voltages are changed. This process repeats until no new neurons spike. We emphasize that in our dynamics we hold the voltage of the neurons that just fired at the resting potential in order to prevent any of these neurons from firing more than once at any given time. After all the neurons are updated at this time, we release these neurons from the reset voltage to follow the dynamics governed by Equation (1) until the next external spike.

### 2.2. Analysis of the I&F network

As is well known, for the balanced state in binary neuronal networks, the population-averaged firing rate is a linear function of the external input. We now address the question of whether the balanced state in the I&F network also possesses the linear response property of the population-averaged firing rate to the external drive.

**Figure 1:**
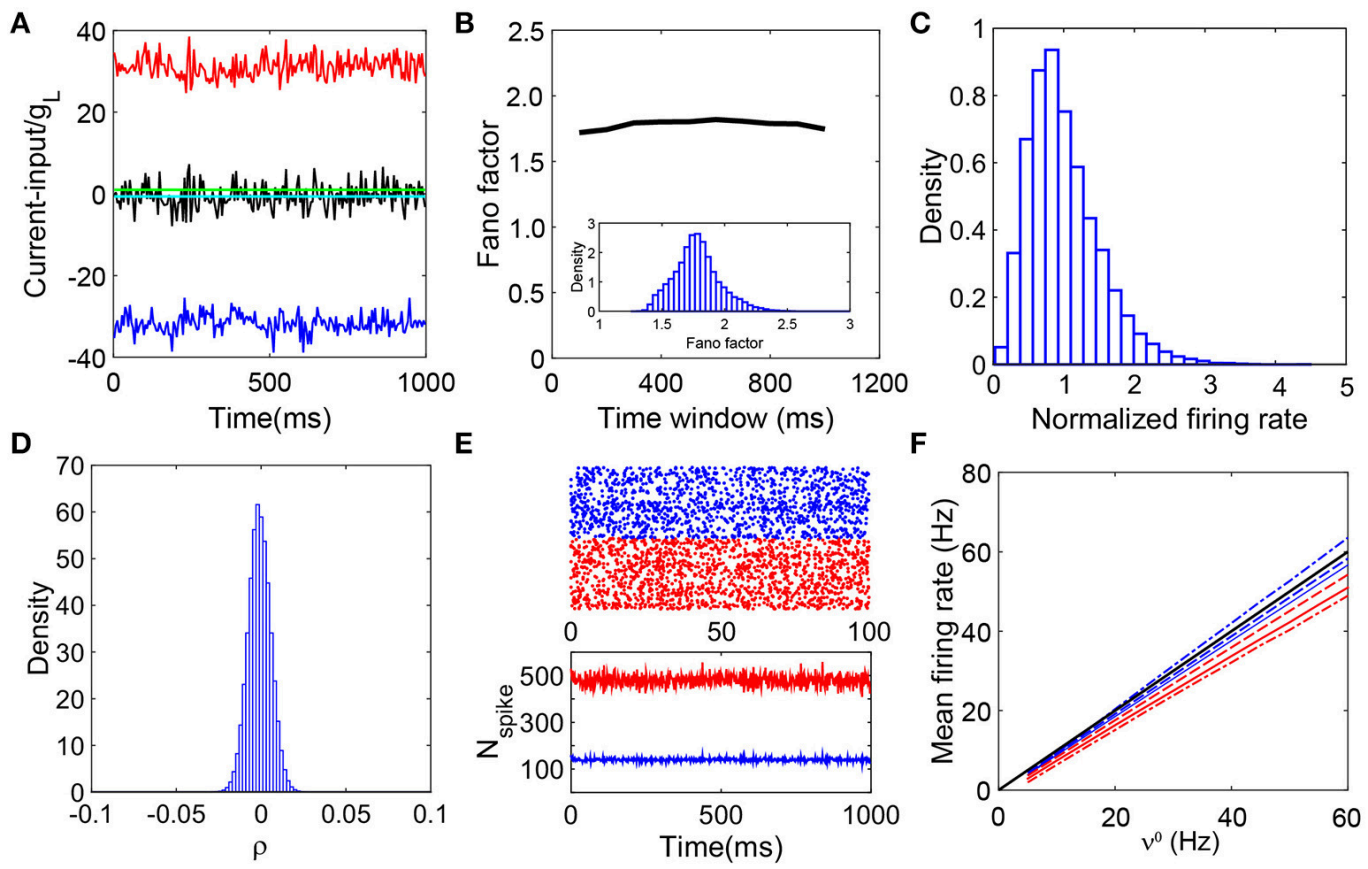
Defining characteristics of a balanced network. **(A)** Balanced excitatory and inhibitory inputs into a sample I&F neuron. The amplitude of excitatory (red) and inhibitory (blue) inputs (normalized by *g*_*L*_) deviate significantly from the firing threshold (green solid line) whereas the summed total input (scaled by *g*_*L*_) (black) fluctuates around the threshold while keeping its mean (cyan) below the threshold; **(B)** The average fano-factor across the network as a function of the time bin size (black line). The values of fano-factor for different time bin sizes (larger than 100 ms) are above unity and nearly constant. The inset is the distribution of the fano-factor calculated from each neurons spike events over the entire network with the time bin size 400 ms. All the values are above unity, again exemplifying the irregularity of the spiking activity; **(C)** Distribution of neuronal firing rates normalized by the entire population-averaged firing rate. The distribution is broad and skewed as a consequence of the heterogeneity of the single neuron activity; **(D)** The distribution of cross-correlation coefficient of spike events for each pair of neurons in the network. It has a sharp peak around zero, therefore, the system is nearly uncorrelated. We choose the bin size Δ*t* = 2ms to calculate the cross-correlation; **(E)** The upper panel is a raster plot of 500 excitatory (red dots) and 500 inhibitory (blue dots) neurons; the lower panel shows the number of the spiking neurons in the excitatory population (red) and inhibitory population (blue) in each time window. The raster plot shows there is no synchrony in the neuronal dynamics. The lower panel exhibits stationarity of the number of firing neurons in the network; **(F)** The mean firing rate of the excitatory and inhibitory population as a linear function of the external driving rate. For comparison, we vary the value of *K* here: *K* = 100 (dashed-dot), *K* = 400 (solid lines), and *K* = 3600 (dashed lines); red for excitatory population whereas blue for inhibitory population. The mean firing rates in the *K* → +∞ limit, $m^{E}=\nu_{0}$ and $m^{I}=\nu_{0}$, are also shown (black line, the theoretical gain curves for the excitatory and inhibitory populations overlap). Note that the minor disagreement between the theoretical prediction and the simulated gain curve arises from the finite size effect, and the gain curve converges to the infinite-*K* limit as *K* increases. The parameters are *N*^*E*^ = 32000, *N*^*I*^ = 8000, $J^{EE}=J^{IE}=1.0/\sqrt{K}$, $J^{II}=1.8/\sqrt{K}$, $J^{EI}=2.0/\sqrt{K}$, $f^{E}=1.0/\sqrt{K}$, $f^{I}=0.8/\sqrt{K}$, ν^*E*^ = ν^*I*^ = ν^0^*K* and ν^0^ = 30Hz. In **(A–E)**, *K* = 400.

We consider the mean firing rate *m*_*E*_ and *m*_*I*_ in the large-*K* limit. In a balanced network, the firing events of different neurons are nearly independent of one another (Vreeswijk and Sompolinsky, 1998). This is illustrated in Figure 1 in which the cross-correlation between firing events of pairs of neurons is narrowly distributed around zero. According to Equations (1) and (2), it is obvious that, under Poisson-train external inputs, spiking events of a neuron in the network, in general, are not a Poisson train, i.e., $\left\{\tau_{js}^{E}\right\}$ and $\left\{\tau_{js}^{I}\right\}$ do not follow Poisson point process for a fixed *j* in Equation (2). However, the input to the *i*th neuron is a spike train summed over outputs from many neurons in the network. Since the firing events of neurons are statistically independent from one another, the summed spike train from a large number of output spike trains of neurons in the network asymptotically approaches a Poisson spike process (Cinlar, 1972). The recurrent excitatory (inhibitory) input of each neuron can be treated as a Poisson train with rate *Km*^*E*^ (*Km*^*I*^), where *m*^*E*^ (*m*^*I*^) is the mean firing rate per neuron averaged over the excitatory (inhibitory) population. Each neuron receives three Poisson spike trains in Equation (2). By homogeneity of our networks, the subscript *i* in Equation (2) will be dropped for the remainder of this discussion.

Since the voltage of neuron in the *k*th population is reset to $\epsilon_{R}^{k}$ after spiking, we consider Equation (1) with initial value $v^{k}\left(0\right)=\epsilon_{R}^{k}$ for *k* = *E, I*. We can readily obtain $v^{k}\left(t\right)=\epsilon_{R}^{k}+f^{k}v^{k,\text{ext}}\left(t\right)+J^{kE}v^{k,E}\left(t\right)-J^{kI}v^{k,I}\left(t\right)$ with $v^{k,l}\left(t\right)=\overset{M^{k,l}}{\underset{s=1}{\sum }}e^{-g_{L}\left(t-U_{s}^{k,l}\right)}$, where *M*^*k,l*^ is the total spike count of the *l*th input to the *k*th population, described by a Poisson distribution with the average number of successes ν^*k,l*^*t*, *k* = *E, I* and *l* = ext, *E, I*. For each given *M*^*k,l*^, the spike times of the *l*th input to the *k*th population, $U_{s}^{k,l}$, *s* = 1, 2, …, are uniformly distributed on the interval [0, *t*]. Clearly, the random variable $R_{s}^{k,l}\left(t\right)\equiv e^{-g_{L}\left(t-U_{s}^{k,l}\right)}$ takes value in the interval $\left[e^{-g_{L}t},1\right]$ with the following probability density $P_{R\left(t\right)}\left(r\right)=\frac{1}{g_{L}t}\frac{1}{r}$ for $r\in \left[e^{-g_{L}t},1\right]$. Since *v*^*k,l*^(*t*) is given by a sum of independent identically distributed random variables for given *M*^*k,l*^, the average neuronal voltage at time *t* can be simply expressed as

(3) $$\begin{array}{r} u^{k}\left(t\right)=\epsilon_{R}^{k}+\frac{1-e^{-g_{L}t}}{g_{L}}\left(f^{k}\nu^{k}+KJ^{kE}m^{E}-KJ^{kI}m^{I}\right) \end{array}$$

for *k* = *E, I*. As discussed above, $\epsilon_{R}^{k}=0$, *f*^*k*^, *J*^*kE*^, and *J*^*kI*^ are of order $1/\sqrt{K}$; ν^*k*^ is of order *K*; and *m*^*E*^ and *m*^*I*^ are of order 1. Then, the leading order of *u*^*k*^(*t*) is $\sqrt{K}$. Obviously, the membrane potential cannot become infinite as *K* → +∞. Therefore, the leading order of $\sqrt{K}$ should vanish and one can obtain

(4) $$\begin{array}{r} \begin{array}{cc} m^{E} & =\frac{J^{II}f^{E}-J^{EI}f^{I}}{J^{IE}J^{EI}-J^{EE}J^{II}}\nu^{0},\\ m^{I} & =\frac{J^{IE}f^{E}-J^{EE}f^{I}}{J^{IE}J^{EI}-J^{EE}J^{II}}\nu^{0} \end{array} \end{array}$$

in the large-*K* limit. Equation (4) describes the linear dependence of the mean firing rate on the external input. This relation is a defining feature of a balanced state, similar to what is obtained for the binary neuronal system (Vreeswijk and Sompolinsky, 1998).

## 3. Results

### 3.1. Existence of balanced states

In Vreeswijk and Sompolinsky (1998), the properties of the balanced state are shown in detail with binary neuronal networks. We use numerical simulations to investigate whether the current-based I&F neuronal network coupled with delta-pulse interactions can exhibit the dynamical characteristics of a balanced state. Our results demonstrate that there indeed exists a balanced state in I&F neuronal networks. Figure 1 summarizes the defining characteristics of the balanced state as exhibited in I&F neuronal networks: *balanced inputs* (Figure 1A), *irregular spiking activity* (Figure 1B), *heterogeneous firing rate* (Figure 1C), *weak correlation* (Figure 1D), *stationary asynchronous dynamics* (Figure 1E), and *linear response* (Figure 1F). The results above confirm the important properties of balanced networks as discussed in previous theoretical work (van Vreeswijk and Sompolinsky, 1996; Vreeswijk and Sompolinsky, 1998; Renart et al., 2010; Litwin-Kumar and Doiron, 2012).

Next, we turn to the investigation of the persistence of the balanced state in the I&F system and derive conditions under which the balanced network state exists. Note that, in our simulation, we choose *J*^*IE*^ = *J*^*EE*^. Therefore, from Equation (4), requiring firing rates to be non-negative implies

(5) $$\begin{array}{r} \frac{f^{E}}{f^{I}}>\frac{J^{EI}}{J^{II}}>1 \end{array}$$

or

(6) $$\begin{array}{r} \frac{f^{E}}{f^{I}}<\frac{J^{EI}}{J^{II}}<1. \end{array}$$

**Figure 2:**
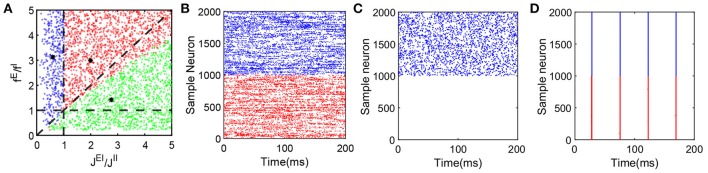
Dynamical network state as a function of *f*^*E*^/*f*^*I*^ and *J*^*EI*^/*J*^*II*^. The range of parameters here is as follows: *N*^*E*^ = 32000, *N*^*I*^ = 8000, *K* = 400, 0 < *f*^*E*^ < 0.2, 0.04 < *f*^*I*^ < 1.0, ν^*E*^*f*^*E*^ = 10, ν^*I*^ = ν^*E*^, $J^{EE}=J^{IE}=1.0/\sqrt{K}$, $1.0/\sqrt{K}<J^{EI}<5.0/\sqrt{K}$, $0.0<J^{II}<10.0/\sqrt{K}$. We choose values of the four parameters *f*^*E*^, *f*^*I*^, *J*^*EI*^ and *J*^*II*^ at random to verify whether the state of the network with these parameters is a balanced state. Note that each dot (*C*_1_, *C*_2_) in the panel **(A)** can represent a group of different parameter values as long as they satisfy $f^{E}/f^{I}=C_{1}$ and $J^{EI}/J^{II}=C_{2}$, where *C*_1_ and *C*_2_ are constant. A red dot represents a balanced state in which both excitatory and inhibitory neurons fire, with a sample raster in panel **(B)** (which has the parameters marked by the black asterisk in the area of red dots, and only 1000 excitatory and 1000 inhibitory neurons are shown here). A green dot represents a state in which only the inhibitory population is firing. This dynamical state also exhibits all the defining characteristics of a balanced state for the inhibitory population, while the excitatory population fires few spikes over a long time. A corresponding sample raster is shown in the panel **(C)** (which has the parameters marked by the black asterisk in the area of green dots, and only 1000 excitatory and 1000 inhibitory neurons are shown here). Finally, a blue dot represents a state of strong synchrony, for which a sample raster is shown in the panel **(D)** (which has the parameters marked by the black asterisk in the area of blue dots, and only 1000 excitatory and 1000 inhibitory neurons are shown here). The black dashed lines in the panel **(A)** are *y* = *x*, *y* = 1 and *x* = 1. In the raster plot, red dots denote the spike times of the excitatory population and blue dots denote the spike times of the inhibitory population.

Note that Equation (5) is equivalent to the balance condition derived in Vreeswijk and Sompolinsky (1998) for a binary neuronal system. However, Equation (6) admits a solution for which *m*^*E*^ = 0 — that is, only inhibitory neurons fire in the system, the mean firing rate of the inhibitory population is then given by *m*^*I*^ = *f*^*I*^ν^0^/*J*^*II*^. Therefore, for *k* = *E* in Equation (3), we can find $u^{E}\left(t\right)\approx \epsilon_{R}^{E}-C\sqrt{K}$, where *C* is a positive constant independent of *K*, which demonstrates that membrane potential is highly negative, and the excitatory neurons' firing activity is suppressed. In the simulation, we choose a range of parameters, in particular, *f*^*E*^, *f*^*I*^, *J*^*EI*^, and *J*^*II*^ to examine the competition between the excitatory and inhibitory inputs to a neuron and verify whether the system maintains the characteristic balanced-state properties. If the neuronal network dynamics indeed exhibits the features displayed in Figure 1, that network is classified as balanced. Figure 2A summarizes our results for the parameters *f*^*E*^/*f*^*I*^ and *J*^*EI*^/*J*^*II*^ that we have scanned. Each dot in the figure can represent a set of parameters *f*^*E*^, *f*^*I*^, *J*^*EI*^, and *J*^*II*^ with fixed ratios of *f*^*E*^/*f*^*I*^ and *J*^*EI*^/*J*^*II*^. Each red dot in the parameter space indicates the fact that the system with the corresponding parameters can reach a balanced state; each blue dot represents the system with the corresponding parameters that exhibits synchronous dynamics; and each green dot indicates that at those parameter values only inhibitory neurons fire in the system but the inhibitory population still retains the characteristic balanced-state properties in Figure 1.

The balanced state with only the inhibitory population firing is established by the balance between strong excitatory external input to the inhibitory neurons and strong inhibitory recurrent current. As shown in Figure 2C, in this state, only the inhibitory population exhibits asynchronous firing activity while the excitatory population is silenced due to relatively strong recurrent inhibition as compared with the inhibitory population.

The parameter values for the results of the representative case as presented in Figure 2B are marked by the black asterisk in the red-dot area in Figure 2A. The parameters for the representative case shown in Figure 2C are marked by the black asterisk in the green-dot area in Figure 2A. The broad region covered by the red and green dots demonstrates the existence of the balanced state over a wide range of parameter space. The parameter values marked by the black asterisk in the blue-dot area in Figure 2A with strong *f*^*E*^ and *J*^*II*^ in comparison to *f*^*I*^ and *J*^*EI*^, respectively, render the system robustly synchronized as shown in Figure 2D.

Note that the area represented by Equation (5) is smaller than the red-dot region that supports stable balanced states in our numerical results. We also point out that the area described by Equation (6) contains a small number of stable balanced states in which both excitatory and inhibitory neurons are spiking in addition to balanced states in which excitatory neurons are almost silent while only inhibitory neurons are spiking. As a matter of fact, upon inserting *m*^*E*^ = 0 and *m*^*I*^ = *f*^*I*^ν^0^/*J*^*II*^ into Equation (3), and requiring that *u*^*E*^ becomes sufficiently negative as *K* becomes large so the excitatory population rarely fires, we obtain the condition

(7) $$\begin{array}{r} \frac{f^{E}}{f^{I}}<\frac{J^{EI}}{J^{II}}, \end{array}$$

which contains Equation (6) and covers a greater area than the parameter regions in Figure 2A filled with green dots. These differences between the numerical results and the theoretical conditions (Equations 5–7) arise from the finite size effect of *K*.

### 3.2. Absence of chaos

As shown above, the I&F networks with delta-pulse coupling can persistently manifest the dynamics of a balanced state. We now address our central question of whether the irregular firing activity of neurons in the balanced network is a consequence of chaotic dynamics of the network. We can mathematically prove that the I&F networks with delta-pulse coupling cannot exhibit chaotic dynamics. Therefore, chaos may not underpin the irregular firing activity of neurons in a balanced state. Below is a proof that there is no chaos in the dynamics of the current-based I&F network coupled with delta-pulses and with a general point process (not limited to Poisson) as external inputs, the details of whose dynamics are described in the section Materials and Methods.

For each reference voltage trajectory, $\text{v}\left(t\right)={(v}_{1}^{E}\left(t\right)$,$v_{2}^{E}\left(t\right),\ldots ,v_{N^{E}}^{E}\left(t\right),v_{1}^{I}\left(t\right)$, $v_{2}^{I}\left(t\right),\ldots ,v_{N^{I}}^{I}\left(t\right))$, we consider the perturbed voltage trajectory $ṽ\left(t\right)={(ṽ}_{1}^{E}\left(t\right),{ṽ}_{2}^{E}\left(t\right),\ldots ,{ṽ}_{N^{E}}^{E}\left(t\right),{ṽ}_{1}^{I}\left(t\right),{ṽ}_{2}^{I}\left(t\right),\ldots$ $,{ṽ}_{N^{I}}^{I}\left(t\right))$, with sufficiently small perturbation size ϵ at initial time *t* = *t*_0_, i.e., ϵ = |***ṽ***(*t*_0_) − ***v***(*t*_0_)| ≪ 1. The dynamics of the perturbed trajectory ***ṽ***(*t*) is described by the equation

(8) $$\begin{array}{l} \frac{d{ṽ}_{i}^{k}}{dt}=-g_{L}\left({ṽ}_{i}^{k}-\epsilon_{R}^{k}\right)+f^{k}\underset{s}{\sum }\delta \left(t-\zeta_{is}^{k}\right)\\ \;+J^{kE}\overset{N^{E}}{\underset{j=1}{\sum }}C_{ij}^{kE}\underset{s}{\sum }\delta \left(t-{\tilde{\tau }}_{js}^{E}\right)-J^{kI}\overset{N^{I}}{\underset{j=1}{\sum }}C_{ij}^{kI}\underset{s}{\sum }\delta \left(t-{\tilde{\tau }}_{js}^{I}\right), \end{array}$$

where ${\tilde{\tau }}_{js}^{k}$ is the *s*th spike of the *j*th neuron in the *k*th population along ***ṽ***(*t*), and *k* = *E, I*. The external spike times $\left\{\zeta_{is}^{k}\right\}$ are the same as those along the reference trajectory **v**(*t*). Then the largest Lyapunov exponent,

(9) $$\begin{array}{r} \lambda_{\text{max}}=\underset{T\to \infty }{\lim}\underset{\epsilon \to 0}{\lim}\frac{1}{T}\ln\;\left(\frac{\left|ṽ\left(T\right)-\text{v}\left(T\right)\right|}{\epsilon }\right), \end{array}$$

can be calculated for this I&F network dynamics (Zhou et al., 2009). As is well known, positive λ_max_ measures the average exponential spreading of nearby trajectories, while negative λ_max_ measures the exponential convergence of trajectories onto the attractor (Oseledec, 1968; Ott, 2002; Parker and Chua, 2012). Generically, an attractor is defined to be non-chaotic if λ_max_ is non-positive. In what follows, we show that the largest Lyapunov exponent λ_max_ of this I&F network is always negative and in fact approaches negative infinity for any spike train input.

#### 3.2.1. Spike train sorting

For the preparation of the proof of the absence of chaos, we label all the neurons as {1, 2, …, *N*^*E*^ + *N*^*I*^}, in which {1, 2, …, *N*^*E*^} labels the excitatory neurons and {*N*^*E*^ + 1, *N*^*E*^ + 2, …, *N*^*E*^ + *N*^*I*^} labels the inhibitory neurons for easy description. That is τ_*js*_ (${\tilde{\tau }}_{js}$) is the *s*th spike of the *j*th neuron, where *j* = 1, 2, …, *N*^*E*^ stands for an excitatory neuron and *j* = *N*^*E*^ + 1, *N*^*E*^ + 2, …, *N*^*E*^ + *N*^*I*^ stands for an inhibitory neuron.

Then for any fixed finite time *T*, we sort the spike times of the two trajectories {τ_*pq*_} and $\left\{{\tilde{\tau }}_{pq}\right\}$ into the increasing lists. Recall the fact that the voltage of both the reference and the perturbed trajectories of any neuron will cross the threshold only upon receiving spikes either from the external or excitatory recurrent input. Hence, the neurons fire precisely at the arrival time of the external or excitatory recurrent input spikes. There may be a group of neurons that fire at a particular time but with a distinct firing sequence amongst these neurons (see our firing dynamics described in the event driven algorithm in section Materials and Methods). Note that these simultaneously firing neurons only spike at the time when an external spike is received by some of the neurons within this group (possibly by all the neurons in the group) for the type of I&F neuronal networks with delta-pulse coupling. When we meet simultaneous firings during the sorting process, suppose these simultaneous firings are τ_*p*_1_*q*_1__, τ_*p*_2_*q*_2__, ···, τ_*p*_*a*_*q*_*a*__ such that τ_*p*_1_*q*_1__ = τ_*p*_2_*q*_2__ = ··· = τ_*p*_*a*_*q*_*a*__, then we perform the following strategy to make the spike timing list unique.

1. *p*_1_ is chosen to be the smallest neuron label among the neurons that receive an external input spike in the simultaneously-firing group;
2. the remaining sequence of τ_*p*_1_*q*_1__, τ_*p*_2_*q*_2__, ···, τ_*p*_*a*_*q*_*a*__ is sorted and reordered such that *p*_2_ < *p*_3_ < ··· < *p*_*a*_.

The same rule is applied in the perturbed network. Then we can obtain two unique increasing spike time sequences

(10) $$\begin{array}{r} \begin{array}{c} \tau_{p_{1}q_{1}}\leq \tau_{p_{2}q_{2}}\leq \cdot \cdot \cdot \leq \tau_{p_{M}q_{M}},\\ {\tilde{\tau }}_{{\tilde{p}}_{1}{\tilde{q}}_{1}}\leq {\tilde{\tau }}_{{\tilde{p}}_{2}{\tilde{q}}_{2}}\leq \cdot \cdot \cdot \leq {\tilde{\tau }}_{{\tilde{p}}_{\tilde{M}}{\tilde{q}}_{\tilde{M}}}, \end{array} \end{array}$$

where τ_*p*_*r*_*q*_*r*__ (${\tilde{\tau }}_{{\tilde{p}}_{\tilde{r}}{\tilde{q}}_{\tilde{r}}}$) denotes the $q_{r}\left({\tilde{q}}_{\tilde{r}}\right)$th spike of the $p_{r}\left({\tilde{p}}_{\tilde{r}}\right)$th neuron in the reference (perturbed) trajectory, $p_{r}\left({\tilde{p}}_{\tilde{r}}\right)=1,2,\ldots ,N^{E}$ stands for an excitatory neuron and $p_{r}\left({\tilde{q}}_{\tilde{r}}\right)=N^{E}+1,$ *N*^*E*^ + 2, …, *N*^*E*^ + *N*^*I*^ stands for an inhibitory neuron. *M* and $\tilde{M}$ are the total number of spikes in the reference and perturbed trajectories, respectively.

#### 3.2.2. Proposition and proof

We next turn to the following proposition,

PROPOSITION *For each reference voltage trajectory* $\text{v}\left(t\right)={(v}_{1}\left(t\right),v_{2}\left(t\right),\ldots ,v_{N^{E}}\left(t\right),v_{N^{E}+1}\left(t\right),v_{N^{E}+2}\left(t\right)$ $,\ldots ,v_{N^{E}+N^{I}}\left(t)\right)\equiv {(v}_{1}^{E}\left(t\right),v_{2}^{E}\left(t\right),\ldots ,v_{N^{E}}^{E}\left(t\right),v_{1}^{I}\left(t\right),v_{2}^{I}\left(t\right),\ldots ,v_{N^{I}}^{I}\left(t\right))$ *described by Equation* (1), *and its perturbed voltage trajectory* $ṽ\left(t\right)={(ṽ}_{1}\left(t\right),{ṽ}_{2}\left(t\right),\ldots ,{ṽ}_{N^{E}}\left(t\right),{ṽ}_{N^{E}+1}\left(t\right),{ṽ}_{N^{E}+2}\left(t\right),\ldots ,{ṽ}_{N^{E}+N^{I}}\left(t\right))\equiv {(ṽ}_{1}^{E}\left(t\right),{ṽ}_{2}^{E}\left(t\right),\ldots ,{ṽ}_{N^{E}}^{E}\left(t\right),{ṽ}_{1}^{I}\left(t\right),{ṽ}_{2}^{I}\left(t\right),\ldots$ $,{ṽ}_{N^{I}}^{I}\left(t\right))$ *described by Equation* (8), *with their initial difference* ϵ = |***ṽ***(*t*_0_) − ***v***(*t*_0_)| *at time* *t* = *t*_0_*, one can obtain the increasing spiking lists* (10) *for both trajectories over any fixed finite time* *T* *according to the sorting process described in the above. If the initial perturbation is sufficiently small, i.e.*, ϵ ≪ 1*, then* $\tilde{M}=M$, ${\tilde{\tau }}_{{\tilde{p}}_{r}{\tilde{q}}_{r}}=\tau_{p_{r}q_{r}}$, ${\tilde{p}}_{r}=p_{r}$ *and* ${\tilde{q}}_{r}=q_{r}$ *for any* *r* *in the lists* (10).

Note that, before the first spike of reference and perturbed trajectories, the time evolution of the perturbation $\delta \text{v}\left(t\right)=\tilde{\text{v}}\left(t\right)-\text{v}\left(t\right)$ can be obtained from the system of equations

(11) $$\begin{array}{r} \frac{d}{dt}\delta v_{i}=-g_{L}\delta v_{i}\left(t\right)\text{ for }i=1,2,\ldots ,N^{E}+N^{I}. \end{array}$$

That is, for a sufficiently small initial perturbation size ϵ, the distance between ***v***(*t*) and ***ṽ***(*t*) decays exponentially. Then we use mathematical induction to prove this proposition:

1. For the first firing event *r* = 1, either τ_*p*_1_*q*_1__ or ${\tilde{\tau }}_{{\tilde{p}}_{1}{\tilde{q}}_{1}}$ is equal to some external input spike time (the first spike must be induced by the external input). Because the reference and perturbed trajectories receive the same external input, for a sufficiently small initial perturbation size ϵ, we have ${\tilde{\tau }}_{{\tilde{p}}_{1}{\tilde{q}}_{1}}=\tau_{p_{1}q_{1}}$, ${\tilde{p}}_{1}=p_{1}$ and ${\tilde{q}}_{1}=q_{1}$;
2. Suppose we have ${\tilde{\tau }}_{{\tilde{p}}_{r}{\tilde{q}}_{r}}=\tau_{p_{r}q_{r}}$, ${\tilde{p}}_{r}=p_{r}$ and ${\tilde{q}}_{r}=q_{r}$ for *r* ≤ *m*, we then show that ${\tilde{\tau }}_{{\tilde{p}}_{m+1}{\tilde{q}}_{m+1}}=\tau_{p_{m+1}q_{m+1}}$, ${\tilde{p}}_{m+1}=p_{m+1}$ and ${\tilde{q}}_{m+1}=q_{m+1}$;
3. In fact, before the (*m*+1)th spike of both the reference and perturbed trajectories, we can find that δ***v***(*t*) is still governed by Equation (11) because they receive the same external and recurrent input spikes. For a sufficiently small initial perturbation size ϵ, Equation (11) also ensures ${\tilde{p}}_{m+1}=p_{m+1}$ and ${\tilde{q}}_{m+1}=q_{m+1}$. Then, if the *q*_*m*+1_th spike of the *p*_*m*+1_th neuron is caused by the arrival of an excitatory recurrent input spike, this spike can always be traced back to the spike that is caused directly by an external input spike. By the ordering rule of neurons in the simultaneously firing group above, we have τ_*p*_*m*+1_*q*_*m*+1__ = τ_*p*_*s*_*q*_*s*__ and ${\tilde{\tau }}_{{\tilde{p}}_{m+1}{\tilde{q}}_{m+1}}={\tilde{\tau }}_{{\tilde{p}}_{s}{\tilde{q}}_{s}}$, where either the spike of τ_*p*_*s*_*q*_*s*__ or the spike of ${\tilde{\tau }}_{{\tilde{p}}_{s}{\tilde{q}}_{s}}$ is caused by an external spike to the *p*_*s*_th neuron (*s* ≤ *m*). Consequently, ${\tilde{\tau }}_{{\tilde{p}}_{m+1}{\tilde{q}}_{m+1}}=\tau_{p_{m+1}q_{m+1}}$. If the *q*_*m*+1_th spike of the *p*_*m*+1_th neuron is caused directly by the arrival of an external spike, clearly we have ${\tilde{\tau }}_{{\tilde{p}}_{m+1}{\tilde{q}}_{m+1}}=\tau_{p_{m+1}q_{m+1}}$ because the reference and perturbed trajectories receive the same external input spike;
4. Finally, we can readily obtain $\tilde{M}=M$.

**Figure 3:**
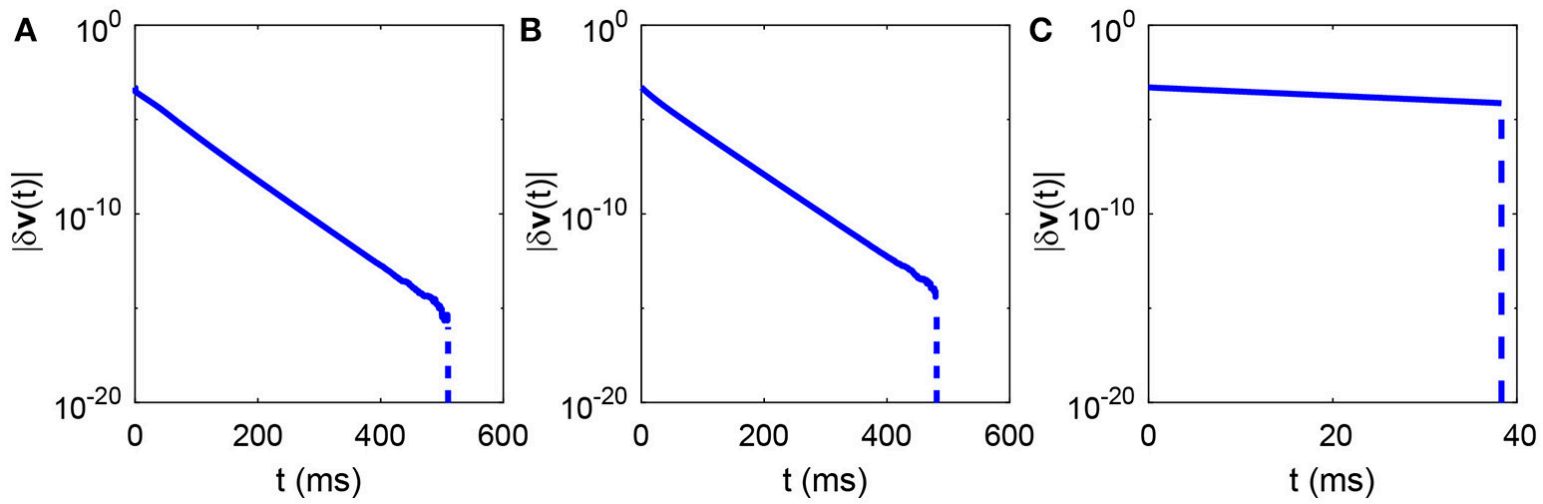
Time evolution of the total perturbation of all neurons' voltages. The total perturbation of all neurons' voltages |δ***v***(*t*)| in either region described in Figure 2A always exponentially decay with time and eventually converge to zero in a finite time. **(A)** The parameters are marked by the black asterisk in the area of red dots in Figure 2A; **(B)** The parameters are marked by the black asterisk in the area of green dots in Figure 2A; **(C)** The parameters are marked by the black asterisk in the area of blue dots in Figure 2A. The parameters are chosen to make the mean firing rate of spiking neurons around 30 Hz. The total perturbation of all neurons' voltages at initial time in each case is chosen to be |δ***v***(0)| = 5 × 10^−4^. The slope of the solid part of the curve in each case is −*g*_*L*_. The vertical dashed part of each curve indicates that the difference between the reference and perturbed trajectories vanishes, namely, the largest Lyapunov exponent approaches negative infinity.

Therefore, ${\tilde{\tau }}_{{\tilde{p}}_{r}{\tilde{q}}_{r}}=\tau_{p_{r}q_{r}}$ holds for *r* = 1, 2, …, *M* with ${\tilde{p}}_{r}=p_{r}$ and ${\tilde{q}}_{r}=q_{r}$. Next, according to the Equation (9), it is obvious that the largest Lyapunov exponent is always negative for the dynamics described by Equation (11). That is, the I&F network with delta-pulse coupling exhibits no chaotic dynamics while receiving pulse-like external input. Note that the voltage of both the reference and perturbed trajectories of any neuron, for example, the *i*th neuron in the *k*th population, will be reset to $\epsilon_{R}^{k}$ after its first firing. Because both the external input spike time and the synaptic input spike time are the same in the reference and perturbed networks, the two trajectories will no longer separate from each other. This means that the difference between the reference and perturbed trajectories of the *i*th neuron will converge after its first firing event. Thus, the largest Lyapunov exponent will approach negative infinity. As show in Figure 3, the numerical results for the given fixed network size *N* and mean degree connectivity *K* indeed show that the total perturbation of all neurons' voltages always exponentially decay with time and eventually converge to zero in a finite time.

As a matter of fact, the proof can be extended to a more generalized system. For example, the membrane potential $v_{i}^{k}$ of the *i*th neuron in the *k*th population obeys equation with refractory periods

(12) $$\begin{array}{l} \frac{dv_{i}^{k}}{dt}=-g_{iL}^{k}\left(v_{i}^{k}-\epsilon_{iR}^{k}\right)+f^{k}\underset{s}{\sum }\delta \left(t-\zeta_{is}^{k}\right)+J^{kE}\overset{N^{E}}{\underset{j=1}{\sum }}C_{ij}^{kE}\underset{s}{\sum }\\ \;\delta \left(t-\tau_{js}^{E}-\tau_{i}^{E}\right)-J^{kI}\overset{N^{I}}{\underset{j=1}{\sum }}C_{ij}^{kI}\underset{s}{\sum }\delta \left(t-\tau_{js}^{I}-\tau_{i}^{I}\right), \end{array}$$

but the neuron will come into a refractory period after $v_{i}^{k}$ crosses the threshold $\epsilon_{iT}^{k}$. That is when $v_{i}^{k}\left(\tau_{is}^{k}\right)\geq \epsilon_{iT}^{k}$, one has $v_{i}^{k}\left(t\right)=\epsilon_{iR}^{k}$ for $\tau_{is}^{k}<t\leq \tau_{is}^{k}+\tau_{i,\text{ref}}^{k}$, where $\tau_{i,\text{ref}}^{k}$ is the refractory period of the *i*th neuron in the *k*th population. $\tau_{i}^{E}$ and $\tau_{i}^{I}$ are the excitatory and the inhibitory synaptic delay of the *i*th neuron, respectively. In this system, the leakage conductance, the threshold, the resting potential, the refractory period and the synaptic delay of one neuron can be different from that of another neuron. Note that a neuron in this system can generate a spike also only upon receiving spikes either from the external or excitatory recurrent input, and the neuron fires precisely either at the arrival time of the external spike or at the arrival time of the excitatory recurrent spike with a fixed synaptic delay. Therefore, we can extend the proof above directly here to conclude that there is no chaotic dynamics in this generalized system. Despite the demonstration of irregular firing of neurons in the balanced state of this current-based I&F network as above, the irregular activity is not induced by chaos.

## 4. Discussion

Cortical neurons often exhibit spiking dynamics that are highly irregular. It is believed that the irregular neural spiking activity can be generated from a balance between excitatory and inhibitory inputs to a neuron. In the early work (Vreeswijk and Sompolinsky, 1998), the neuronal network consisting of binary neurons was applied to explain the balanced neuronal state. Meanwhile, the dynamics of the binary neuronal system was found to be chaotic in the balanced state. Therefore, chaos was then often thought to be closely related to the irregular firing activity in a balanced network. Note that the binary neuron is a highly idealized model for describing a neuron.

Here we address the issue of chaotic origin of spiking irregularity using a current-based leaky integrate-and-fire (I&F) model with delta-pulse coupling and pulse-like external input. By examining the defining characteristics of a balanced state (Vreeswijk and Sompolinsky, 1998), and by exploring a wide range of parameter values, we have found that the balanced state persists robustly in this I&F system.

We then apply the analysis of the largest Lyapunov exponent to characterize the dynamics of this I&F system. We mathematically demonstrate that the largest Lyapunov exponent is always negative in the current-based I&F system with delta-pulse coupling and pulse-like external input, in which each neuron can have its own distinct values of leakage conductance, resting potential, spiking threshold, refractory period, and synaptic decay. The reference and perturbed realizations of a neuronal network trajectory can converge in finite time. It is worthwhile to point out that the non-chaotic property in our proof holds for neuronal networks of any size.

To understand under what conditions cortical-like firing irregularity can be generated, several efforts have been made. For instance, an early study (Renart et al., 2007) investigated the possible existence of multiple balanced steady states with persistent activity. It shows that the Poisson-like irregular spiking activity can arise from balanced regimes with sustained persistent activity. The neuronal network system used in the study (Renart et al., 2007) is the same as that in our work, but with different scaling of synaptic connection strengths, e.g., homogeneous or heterogeneous multicolumnar architecture. Note that the proof of non-chaotic mechanism in our system is independent of scaling structure of connection strengths as long as the synaptic interaction is delta-pulse coupled. Therefore, it is expected that the non-chaotic mechanism also works in persistent activity states.

In addition, another recent study found that the I&F neuronal network endowed with probabilistic synaptic transmission can underlie the Poisson-like spiking variability over a wide range of firing rates (Moreno-Bote, 2014). Here, we show that the neuronal network consisting of I&F neurons with delta-pulse couplings is always non-chaotic over a wide range of firing rates. It could be interesting to investigate whether our non-chaotic neuronal network with probabilistic synaptic transmission can achieve Poisson-like variability of the spiking activity as observed in cortex. For example, we can consider that each pair of connected neurons in our system has multiple delta-pulse synaptic contacts, and each contact is activated with certain probability.

As is shown here in our works, the irregular spiking dynamics is always stable in the current-based I&F model with delta-pulse coupling and pulse-like external input. Therefore, the irregular activity cannot simply arise from the underlying chaotic dynamics. We point out that the proof of the non-chaotic dynamics in our system relies on the facts that both the external input and the synaptic interactions are in the form of delta-pulse coupling. Therefore, for any fixed network size *N* and the mean degree connectivity *K*, the neuron fires precisely at the arrival time of the external or excitatory recurrent spikes. As a result, the difference between the reference and perturbed trajectories of each neuron will converge after its first firing event. Thus, the largest Lyapunov exponent will approach negative infinity and the dynamics is non-chaotic. As for the case of smooth synaptic coupling, e.g., the current $I_{i}^{k}\left(t\right)$ in Equation (2) is an α-like function (Dayan and Abbott, 2001) with the rise time constant τ_*r*_ and the decay time constant τ_*d*_ as $I_{i}^{k}\left(t\right)={(e}^{-t/\tau_{r}}-e^{-t/\tau_{d}})/\left(\tau_{r}-\tau_{d}\right)$, it is expected that the non-chaotic mechanism still holds when the time constants τ_*r*_ and τ_*d*_ are both relatively small. However, if these time constants are not sufficiently small, e.g., greater than 2 ms, it is known that the network dynamics is not always stable and can be chaotic (Zhou et al., 2010; Harish and Hansel, 2015). In general, the phenomenon of chaos is model-dependent (Brette, 2004; Zhou et al., 2009; Sun et al., 2010) and could not be the ultimate source of irregularity in neuronal activity in the brain.

## Author contributions

QG, ZT, GK, DZ, and DC: Conceived and designed the research, Performed experiments and analyzed data, Wrote the paper.

### Conflict of interest statement

The authors declare that the research was conducted in the absence of any commercial or financial relationships that could be construed as a potential conflict of interest.
